# Cardiovascular Risk Awareness and Calculated 10-Year Risk Among Female Employees at Taibah University 2019

**DOI:** 10.3389/fpubh.2021.658243

**Published:** 2021-10-04

**Authors:** Amal M. Qasem Surrati, Walaa Mohammedsaeed, Ahlam B. El Shikieri

**Affiliations:** ^1^Family and Community Medicine Department, College of Medicine, Taibah University, Medina, Saudi Arabia; ^2^Medical Laboratories Technology Department, College of Applied Medical Sciences, Taibah University, Medina, Saudi Arabia; ^3^Clinical Nutrition Department, College of Applied Medical Science, Taibah University, Medina, Saudi Arabia

**Keywords:** cardiovascular disease, knowledge, awareness, risk factors, Madinah-KSA, calculated 10 year risk

## Abstract

Cardiovascular diseases (CVD) are the most common cause of death and disability worldwide. Saudi Arabia, one of the middle-income countries has a proportional CVD mortality rate of 37%. Knowledge about CVD and its modifiable risk factors is a vital pre-requisite to change the health attitudes, behaviors, and lifestyle practices of individuals. Therefore, we intended to assess the employee knowledge about risk of CVD, symptoms of heart attacks, and stroke, and to calculate their future 10-years CVD risk. An epidemiological, cross-sectional, community-facility based study was conducted. The women aged ≥40 years who are employees of Taibah University, Al-Madinah Al-Munawarah were recruited. A screening self-administrative questionnaire was distributed to the women to exclude those who are not eligible. In total, 222 women met the inclusion criteria and were invited for the next step for the determination of CVD risk factors by using WHO STEPS questionnaire: It is used for the surveillance of non-communicable disease risk factor, such as CVD. In addition, the anthropometric measurements and biochemical measurements were done. Based on the identified atherosclerotic cardiovascular disease (ASCVD) risk factors and laboratory testing results, risk calculated used the Framingham Study Cardiovascular Disease (10-year) Risk Assessment. Data were analyzed using GraphPad Prism 7 software (GraphPad Software, CA, USA). The result showed the mean age of study sample was 55.6 ± 9.0 years. There was elevated percentage of obesity and rise in abdominal circumference among the women. Hypertension (HTN) was a considerable chronic disease among the participants where more than half of the sample had it, i.e., 53%. According to the ASCVD risk estimator, the study participants were distributed into four groups: 63.1% at low risk, 20.2% at borderline risk, 13.5% at intermediate risk, and 3.2% at high risk. A comparison between these categories based on the CVD 10-year risk estimator indicated that there were significant variations between the low-risk group and the intermediate and high-risk groups (*P* = 0.02 and *P* = 0.001, respectively). The multivariate analysis detected factors related to CVD risk for women who have an intermediate or high risk of CVD, such as age, smoking, body mass index (BMI), unhealthy diet, blood pressure (BP) measurements, and family history of CVD (*P* < 0.05). The present study reports limited knowledge and awareness of CVD was 8.6 that is considered as low knowledge. In conclusion, the present study among the university sample in Madinah reported limited knowledge and awareness of CVD risk. These findings support the need for an educational program to enhance the awareness of risk factors and prevention of CVD.

## Introduction

Cardiovascular disease (CVD) is coronary heart disease (CHD), heart failure, ischemic stroke, peripheral vascular disorder, and atherosclerosis of the aorta and its branches. The lifetime risk of CVD reaches 50% for those aged 30 years without known CVD (1). Many countries in the Gulf Cooperation Council have suffered a higher number of deaths from non-communicable diseases which are estimated to comprise 65–78% of the total adult loss (2) and ~73% of all death in Saudi Arabia (2016) (3). The main changeable risk factors for CVD are dyslipidemia, type 2 diabetes (T2DM), increased blood pressure (BP), obesity, and smoking, and these are claimed to be responsible for more than 50% of cardiovascular mortality (4). Using data from 52 countries, the INTERHEART study found that nine changeable factors accounted for more than 90% of the risk of new-onset myocardial ischemia: tobacco, dyslipidemia, hypertension (HTN), diabetes, central obesity, psychosocial factors, daily intake of fruits and vegetables, regular alcohol consumption, and frequent physical exercise (5). Persistent exposure to harmful factors causes a greater increase of atherosclerotic plaque.

Risk calculation for CVD is an important method to predict the future impact of atherosclerotic cardiovascular disease (ASCVD) and to enhance the adherence to healthy lifestyle measures and therapies (6). The use of risk calculators is intended to predict total CVD risk depending on the presence of independent factors in a mathematical equation, which concludes that a percentage depends on the absolute risk for other outcomes. This allows the physician to calculate the risk to prescribe therapy of each client. More than 100 scores were available in the literature. CVD risk calculation measures are approved to use for adults to guide therapy for hyperlipemia, HTN, and diabetes. For example, the Framingham Risk Assessment is an easy and validated method for recognizing persons at risk for ASCVD (7).

The result of the Framingham research showed that for individuals of 50 years of age with no cardiovascular problems, the risk of harmful effects happening later in life was 51.7% in men and 39.2% in women, with a median survival of 30 and 36 years, respectively. Estimation of future risk is important in adulthood because, if attention is paid only to short-term risk, fewer people will modify their daily habits by obeying a management plan (8). Most of the risk factors for CVD and stroke are changeable with protective strategies, such as therapeutic lifestyle changes (TLCs) and the introduction of therapy with proven benefits (9). With primary prevention, the modification of multiple major risk factors will further reduce the risk of coronary artery disease (CAD) and stroke (10). The high-risk category is defined as adults with a 10-year risk of ASCVD >7.5% or a lifetime risk >30%, as well as those with very high low-density lipoprotein (LDL) cholesterol (LDL-C) levels or primary genetic hyperlipidemias for whom equations may not correctly reflect the risk. The 2013 American Heart Association (AHA)/American College of Cardiology (ACC) guidelines recommended the treatment with a statin for patients with a 10-year risk of ≥7.5%, although the statin treatment can be considered even at a risk of 5% (11). Primary prevention, healthy lifestyle strategies to help high-risk individuals decrease their CVD risk include: stopping, reducing, or avoiding smoking; maintaining a healthy diet; being physically active; keeping a body mass index (BMI) <25 kg/m^2^ and a waist-hip ratio <0.8 in women and <0.9 in men; lowering BP to <140/90 mmHg; lowering TC to <190 mg/dl; decreasing LDL-C to <115 mg/dl; targeting the glucose levels in patients with impaired fasting glucose (IFG), impaired glucose tolerance (IGT), or diabetes; and prescribing aspirin at 75 mg/d if BP is controlled.

Many countries have adapted primary prevention measures to decrease the later costs of complicated cases requiring tertiary care. The key aspect leading to the proven effectiveness of this approach is the perception of the risk of an individual for certain health diseases (12). In any sector considered an important representative sample, workers' quality of life, health beliefs, and ability to adapt healthy measures are predicted to affect their productivity and likelihood of avoiding chronic disease. By decreasing the healthcare demand, the financial status of any institution will improve (13). Awareness of CVD and its modifiable risk factors is an important step toward modulating the health beliefs, behaviors, and daily habits of individuals (14, 15). It also helps them correctly calculate their future risk and motivates them to follow prevention-seeking actions and adapt behavior to decrease harm (16–18). Furthermore, estimating the current level of information about CVD available in a community has positive public health effects as it helps in creating focused educational materials as well as planning (19).

Knowledge about CVD and its modifiable risk factors is a vital prerequisite to the changing health attitudes, behaviors, and lifestyle practices of individuals. As far as we know, there is limited quantitative data focusing on awareness and knowledge of CVD risk factors in Madinah city. Therefore, we aimed to determine the cardiovascular risk awareness and calculated 10-year risk among the women aged ≥40 years.

## Materials and Methods

A descriptive, cross-sectional, community-based study was conducted between March 1 and August 30, 2019, among the women aged 40 years and above who are current employees at Taibah University.

### Data Collection Method and Tools

The required sample size was calculated to be 385 participants. The calculation was done using the sample size software online (available at http://sampsize.sourceforge.net/iface/#prev) for prevalence studies assuming unknown exactly prevalence of CVD risk awareness 50% at *CI* 95% and power of test 80%.

A self-administered screening questionnaire developed by the researchers was distributed to the women to eliminate those who were not eligible. Those women who fulfilled the inclusion criteria were selected, and data were collected from them in a systematic, non-invasive way. Participants with complete data and had their blood samples drawn were included in the study (*n* = 222). The exclusion criteria covered individuals who had been diagnosed with CVD, such as myocardial ischemia, stroke, unstable angina, revascularization surgery, disease of the aorta, coronary angioplasty, and peripheral arterial disease, as well as pregnant women.

They were told the objective of the study and were free to choose not to participate. Confidentiality was guaranteed, and they were given an electronic agreement to sign. The questionnaire included the selected demographic and health information, such as the age of the participant and the presence of any health problems. They were then invited to participate in the next step when CVD risk factors were determined. The tools used in this stage are outlined below. The questionnaire was pre-tested before the start of the study to ensure their accuracy and precision. The tools were pretested for clarity and understanding on 10 volunteers and adjustments were carried out accordingly.

A modified WHO STEPS instrument, which is used for the surveillance of non-communicable disease risk factors that include the risk factors associated with CVD, was employed. The questionnaire was accessed via the weblink www.who.int/ncds/steps. The sociodemographic characteristics (e.g., age and education level) and behavioral measurements (e.g., tobacco use; dietary pattern; physical activity level; a history of diabetes, HTN, hyperlipidemia, and CVD; lifestyle advice; and cervical cancer screening) of the participants were determined. Information such as marital status, education level, and occupation was recorded.

Dietary data were assessed using 2-weekdays 24-h recall records. Foods were converted into nutrients using the Diet Organizer application version 3.1 (MulberySoft, Thailand), and results were compared with recommended dietary allowance (RDA) standardized based on 40–51 and 52–63 years. The intake of certain foods that were previously reported to be associated with CVDs risk was also assessed. These included foods, such as the intake of whole grain cereals, high fiber foods, fruit, and vegetables. In this study, the number of servings for fruits, vegetables, and water intake were obtained from the Saudi Healthy Food Palm (fruit serving = 2–4/day, vegetables serving = 3–5/day, and water at least 6 cups/day).

The physical evaluations were performed, measuring BP ≥140 systolic and /or ≥90 diastolic consider hypertensive, pulse, and anthropometric analysis (such as weight, height, and waist circumference [WC]) calculated as below.

Weight was measured two times following published protocols using OMRON—Body Fat Scales (BF508l, China) after being calibrated. The WC was measured two times using a non-stretchable measuring tape, and for accuracy, the readings were taken from the right side of the body (two fingers above the navel). The risk of CVD was increased in women with WC >88 cm. BMI was calculated using standard formulas (weight in kilogram and height in meters square) for those ≥30 consider obese.

The biochemical measurements were analyzed following the published protocols. These included lipid profile and fasting blood glucose. All tests were performed in clinical biochemistry labs in the National Guard Hospital (the kits and lab items were supported by Taibah University), using an automated machine (ARCHITECT c4000, Abbott Laboratories, IL, USA).

Build on the recognized ASCVD risk factors and laboratory testing results, ASCVD risk was approximated using the Framingham Study Cardiovascular Disease (10-year) Risk Assessment (9) (validated in the age of 30+ population) and the ASCVD Risk Estimator for both 10-year and lifetime ASCVD risk (validated in the age 40–79 years and age 20–60 years populations, respectively). Using an online risk calculator (https://tools.acc.org/ldl/ascvdriskestimator/index.html/calculate/estimator), the participants were categorized into four risk groups based on the 10-year ASCVD risk percentages: low risk [ <5%], borderline risk [5%– <7.5%], intermediate-risk [>7.5%– <20%], and high risk [>20%].

The total CVD knowledge score was calculated as a continuous variable by summing the scores of the respondents concerning CVD types, heart attack symptoms, stroke symptoms, and CVD risk factors. The total knowledge score was 24, and the levels of CVD knowledge were categorized as low knowledge [ ≤ 12], moderate knowledge (13–19), and high knowledge [≥20] (20). Each correct response was given a score of 1, and a wrong response was scored as 0, making the total score 24 with each subdivision having a score of between 4 and 9. Knowledge results were classified for each part as follows:

- Stroke symptoms (Score = 5) Factors of CVD (Score = 9)
- CVD symptoms (Score = 6) Heart attack symptoms (Score = 4)

Ethical permission was sought from the Ethical Clearance Committee at the Faculty of Applied Medical Sciences. SREC/AMS 2019/42/CND on 22-Oct-2019 Permission was also obtained from the colleges, committees, and sectors from where the samples were recruited. The written consent forms were signed by all the participants before the start of the study, affirming that all data would be treated with confidentiality and that they have the right to withdraw from the study at any time they wished.

### Operational Definitions of the Variables Used in Study

1- Body mass index is the ratio between weight in kilograms to height in meters squared. Based on the National Institutes of Health (NIH), BMI of the participants was classified as underweight (BMI ≤ 18.0), normal (BMI = 18.5–24.9), overweight (BMI = 25.0–29.9), or obese (BMI ≥30.0).
2- Blood pressure was measured by an automatic device. The measurements were taken in the right upper limb, after 5 min of rest, with the subject seated and the arm supported. BP was recorded as the average of three measurements taken 5 min apart during rest. The average of the three BP measurements was calculated. Normal systolic blood pressure is defined as <140 mmHg and diastolic <90 mmHg.
3- Waist circumference was measured two times using a non-stretchable measuring tape, and for accuracy, the readings were taken from the right side of the body (two fingers above the navel). The CVDs risk was increased in women with WC >88 cm.
4- Lipid panel measurement an elevated cholesterol is defined as a total cholesterol ≥200 mg/dl and a low HDL level as <40 mg/dl an elevated low-density lipoprotein (LDL) is defined per ACC/AHA guidelines as a primary elevation ≥190 mg/dl or an LDL of >70 mg/dl with the presence of diabetes in a subject 40–75 years of age, and patients with a ASCVD predicted risk >7.5% with an LDL >70 mg/dl. Triglyceride levels is defined as ≥200 mg/dl.

### Statistical Analysis

Data were analyzed using the GraphPad Prism 7 software (GraphPad Software, CA, USA). Quantitative data were expressed as frequency (percentages) and mean ± SD. The Student's *t*-test was used to compare four categories of calculated CVD risk. A multivariable statistical analysis (multiple logistic regression model) was performed to study the association between the 10-year CVD risk factors and knowledge of CVD based on the chosen factors. Data were presented with a 95% *CI* and an odds ratio (*OR*).

## Results

### Study Population: Demographic and Clinical Characteristics of Study Participants

The demographic and clinical characteristics of the 222 women included in the study were as follows: their mean age was 55.6 ± 9.0 years, 81% of them were married, and 47.7% had a bachelor's degree. The BMI mean of the study population was 29.9 ± 5 kg/m^2^, and 34% of them were overweight with 44.6% considered obese. Abnormal waist circumference >88 cm was seen in 63% of them, and 33.8% had a high and 28.3% very high percentage of body fat. More than half of the women, 58%, had an appropriate sleep duration of 6–8 h/day while a short sleep duration of fewer than 5 h/day was reported by 37% of the samples (assessed from a self-administered questionnaire in WHO STEPS). Unhealthy dietary habits were seen in 85% of the participants. HTN was the most common chronic problem, present in 53%, followed by diabetes in 7%. The most frequently reported problems in the family history of the samples were hypercholesterolemia in 23% and HTN in 18% (all data evaluated from the self-administered questionnaire in WHO STEPS) (Table 1).

**Table 1:** Demographic and clinical characteristic of women at Taibah University (*n* = 222).

**Variables**	**Number of women (%)**
**Age**	
Mean ± SD	55.6 ± 9.0
40–51 years	184 (82.9%)
52–63 years	38 (17.1%)
**Marital status**	
Single	18 (8.1%)
Married	179 (80.6%)
Divorced	20 (9.1%)
Widow	5 (2.3%)
**Education level**	
Primary complete and below	33 (14.9%)
Secondary and higher schooling	38 (17.1%)
Complete university or college	106 (47.7%)
Post-graduate studies	45 (24.6%)
**Smoking status**	
Smoker	22 (9.9%)
Non-smoker	200 (90.1%)
**BMI classification**	
Normal	47 (21.2%)
Overweight	76 (34.3%)
Obesity grade I	63 (28.4%)
Obesity grade II	25 (11.3%)
Obesity grade III	11 (4.9%)
**Average waist circumference (cm)**	
Mean ± SD	90.1 ± 43.3
<88.0 cm	82 (36.3%)
>88.0 cm	140 (63.7%)
**Classification of percent body fat**	
Low <15%	15 (6.8%)
Normal (18–22%)	25 (11.3%)
Moderately (22–30%)	44 (19.8%)
High (30–40%)	75 (33.8%)
Very high >40%	63 (28.3%)
**Sleeping pattern (hours/day)**
Appropriate sleep (6–8 h/d)	128 (57.7%)
Short sleep (≤ 5 h/d)	82 (36.9%)
Long sleep (≥9h/d)	12 (5.4%)
**Categories of blood pressure**	
Normal	89 (40.1%)
Elevated	16 (7.2%)
Stage 1	74 (33.3%)
Stage 2	42 (18.9%)
Hypertensive crisis	1 (0.5%)
**An unhealthy diet**	
Unhealthy diet	189 (85.1%)
Healthy diet	33 (14.9%)
**Family's history of disease**	
Diabetes	13 (5.9%)
Die at an early age due to CVD	32 (14.4%)
CVD	23 (10.4%)
Hypertension	42 (18.9%)
Hypercholesterolemia	52 (23.4%)
**Chronic disease**	
Diabetes	16 (7.2%)
Hypertension	117 (52.7)
Hyperlipemia	5 (2.3%)
Chronic renal disease	2 (0.9%)
Lupus	2 (0.9%)
Rheumatoid arthritis	2 (0.9%)
Psoriasis	4 (1.8%)
**Blood analysis**	
FBG (mmol/L)	4.9 ± 1.19
Total cholesterol (mmol/L)	**6.2** **±** 1.1*
LDL-cholesterol (mmol/L)	**3.1** **±** 0.84*
HDL-cholesterol (mmol/L)	1.4 ± 0.37
Triglycerides (mmol/L)	**1.91** **±** 1.1*

The results presented as frequency (percentage %). The high levels of certain biomarkers, such as cholesterol, LDL, and triglycerides significantly beyond the normal range in healthy women are marked with bold and an asterisk (*) on the table and the data presented as the mean concentrations ± SD. FBG, fasting blood glucose; LDL, low density lipoprotein; HDL, high density lipoprotein.

### Calculated 10-Year Cardiovascular Risk Among Women at Taibah University

According to the online ASCVD risk estimator, the study participants were divided into four categories: 63.1% at low risk, 20.2% at borderline risk, 13.5% at intermediate risk, and 3.2% at high risk (Table 2). A comparison among these categories according to the CVD 10-year risk estimator indicated that there were significant differences between the low-risk group and the intermediate and high-risk groups (*P* = 0.02 and *P* = 0.001, respectively).

**Table 2:** Cardiovascular calculated 10-year risk among female at Taibah University.

**CVD calculated risk categories**	**Frequency (%)**	**Mean ± SD**	***P*-value**
Low (<5%)	140 (63.1%)	3.9 ± 0.67	
Borderline (5–7.4%)	45 (20.2%)	6.82 ± 0.88	0.06
Intermediate (7.5–19.9%)	30 (13.5%)	15.5 ± 1.42	0.02*
High (>20%)	7 (3.2%)	21.3 ± 2.4	0.001**

Data presented as frequency (percentage %) and mean ± SD. The t-test was used to compare between low group and the other groups. Statically significant at *P ≤ 0.05 or **P ≤ 0.001.

### Respondents' Knowledge of CVD Types, Heart Attack, and Stroke Symptoms, and CVD Risk Factors for Women

Table 3 indicates that the most frequent type of CVD reported was CHD [39.6%] followed by rheumatic heart disease [28.8%] and congenital heart disease [27.5%]. The most common stroke symptoms appearing in collected data were sudden dizziness/difficulty walking [62%], sudden lack of focus/difficulty speaking [60%], sudden numbness/weakness in the face or arms [55%], and sudden visual impairment [43%]. For heart attack symptoms, the respondents reported chest pain/discomfort [78%], shortness of breath [74%], and pain in the arms or shoulders [55.7%]. Participants indicated knowledge of the following CVD risk factors: unhealthy diet [85%], hypertension [80%], obesity [79%], smoking [77%], hypercholesterolemia [75%], lack of exercise [71%], stress/anxiety [67%], and diabetes [61%]. Each correct response was given a score of 1, and a wrong response was scored as 0. The total possible score was 24, the knowledge scores were categorized as follows: stroke symptoms (Score = 5), heart attack symptoms (Score = 4), risk factors of CVD (Score = 9), and CVD diseases (Score = 6). Calculated scores indicated that the overall knowledge of the participants was 8.6 which was considered low knowledge as presented in Table 3.

**Table 3:** Percentage of women giving correct response to CVD risk factors and their response scores.

**Variables**	**Percentage of women (%)**	**Mean score**
**Stroke symptoms (Score** **=** **5)**		
Severe headache without a known cause	37.8%	1.4
Sudden dizziness, difficulty walking, or loss of balance	62.6%	2.3
Sudden visual impairment in one or both eyes	42.8%	1.5
Sudden lack of focus, difficulty speaking, or understanding others	60.4%	2.2
Sudden numbness/weakness in the face, arm, or leg	55.4%	2.1
The total score		9.5/5 = 1.9
**Heart attack (Score** **=** **4)**		
Shortness of breath	73.9%	2.7
Pain in the arms or shoulder	55.7%	2.1
Chest pain or discomfort	78.4%	2.9
Pain in the neck, jaw, or back	40.4%	1.5
The total score		9.2/4 = 2.3
**Factors of CVD (Score** **=** **9)**		
An unhealthy diet	85.1%	3.2
Obesity	78.8%	2.9
Stress and anxiety	67.6%	2.5
Lack of exercise	70.7%	2.6
Harmful increase in cholesterol	75.3%	2.8
Hypertension	79.7%	3
Smoking	77.1%	2.8
A family history of cardiovascular disease	49.5%	1.8
Diabetes	61.3%	2.3
The total score		23.9/9 = 2.7
**Considered as CVD diseases symptoms (Score** **=** **6)**		
Lung arterial thrombosis	26.6%	1.9
Congenital heart disease (defects from birth)	27.5%	1.0
Heart rheumatism	28.8%	1.1
Vascular stenosis disease	18.9%	1.6
Brain arterial disease	8.6%	2.9
Coronary heart disease	39.6%	1.5
The total score		10/6 = 1.7
The total knowledge score = 24		8.6

*Data presented as percentage (%). The overall cardiovascular disease (CVD) knowledge score was calculated as a continuous variable by summing the respondent's scores of CVD risk factors. The maximum overall knowledge score was 24: low knowledge ≤ 12, moderate knowledge = 13–19, and high knowledge ≥20*.

### Factors Associated With 10-Year CVD Risk Estimates Among Women

The multivariate analysis identified factors associated with CVD risk for women who have an intermediate or high risk of CVD (*n* = 37). As demonstrated in Table 4, factors independently related to CVD risk were age, smoking, BMI, unhealthy diet, blood pressure measurements, and family history of CVD (*P* < 0.05). The CVD risk was significantly greater among the women aged 52–63 years compared with other age groups (*P* = 0.02). The study participants were found to be at a higher CVD risk if they reported having a high BMI, having a high BP level (*OR* = 6.8), regularly eating an unhealthy diet (*OR* = 3.8), and having a family history of CVD (*OR* = 3.7). There was also a strong association between increased risk of CVD and the levels of lipids, such as cholesterol (*OR* = 7.8, *P* = 0.001) and LDL (*OR* = 6.9, *P* = 0.003, Table 4).

**Table 4:** Factors associated with 10-year CVD risk estimate among women with intermediate and high risk (*n* = 37).

**Variables**	**Number of women (%)**	**OR**	**95% CI**	***P*-value**
**Age**				
40–51 years	20 (54.1%)	Reference		0.02*
52–63 years	17 (45.9%)	3.81	1.533–4.712	
**Marital status**				
Single	10 (27%)	1.8	0.876–1.987	>0.05
Married	27 (73%)	Reference		
**Education level**				
Secondary and higher schooling	11 (29.7%)	Reference		>0.05
Complete university or college	26 (70.3%)	1.5	1.992–1.765	
**Smoking status**				
Smoker	12 (32.4%)	4.6	0.998–5.871	0.01*
Non-smoker	25 (67.6%)	Reference		
**BMI classification**				
Normal	5 (13.5%)	Reference		
Overweight	13 (35.2%)	4.7	2.651–6.876	0.03*
Obesity	19 (51.3%)	5.8		0.002**
**Categories of blood pressure**				
Normal	16 (43.2%)	Reference		
Elevated	16 (43.2%)	1.9	0.654–1.987	>0.05
Stage 1	5 (15.6%)	6.8	2.651–7.976	0.01*
**An unhealthy diet**				
Unhealthy diet	27 (72.9%)	3.8	1.761–4.951	0.04*
Healthy diet	10 (27.1%)	Reference		
**Family's history of disease**				
Yes	21 (56.8%)	3.7	1.555–4.982	0.05*
No	16 (43.2%)	Reference		
**Blood analysis**				
FBG (mmol/L)	3.8 ± 1.33	1.9	0.896–1.996	>0.05
Total cholesterol (mmol/L)	6.92 ± 1.8*	7.8	2.621–9.811	0.001**
LDL-cholesterol (mmol/L)	3.7 ± 0.97*	6.9	1.842–7.861	0.003**
HDL-cholesterol (mmol/L)	1.7 ± 0.67	1.7	0.643–0.898	>0.05
Triglycerides (mmol/L)	1.88 ± 1.31	1.5	0.652–1.943	>0.05

Multiple logistic regression showed odd ratios (OR), with a 95% CI values for the factors associated with CVD risk. All differences were statistically significant at a level of *P ≤ 0.05 or **P ≤ 0.001. The analysis was done to evaluate the most risk factors between two groups.

The results of the multivariate analysis for factors associated with total knowledge of CVD scores and the characteristics of respondents. In the multivariate logistic analysis, factors significantly associated with CVD knowledge included are age, education, BP measurements, healthy diet, BMI, and family history of CVD (*P* < 0.05). Knowledge about CVD was markedly greater among the women aged 40–51 years in contrast to other age groups (*P* = 0.001, data not shown). The study participants were found to be more knowledgeable about CVD if they had reported high education levels (*P* < 0.05), a consistently healthy diet (*P* = 0.003), a normal BMI (*P* = 0.03), and a family history of CVD (*P* = 0.01).

## Discussion

Our study aimed to determine the level of knowledge about CVD risk factors, heart attacks, and stroke symptoms among the women and to calculate their risk, meaning the probability of an individual experiencing a CVD event over a given period. Our study results identified the knowledge gaps among our community regarding the CVD risk, and therefore would help in planning future health educational programs while addressing the prevalent needs.

Although people usually underestimate their CVD risk, the possibility of realizing that they are at a higher risk increases when they are aware of the presence of a risk factor (21). Anticipating future risks helps physicians apply primary preventive measures, such as providing health education for the person and community at risk before complications occur. The WHO member states have committed to providing counseling and therapy for at least 50% of the people aged 40 years or older who have a high risk of CVD by 2025 (22). Most individuals in our sample were obese 45%, and 34% were overweight. In comparison, 29.4% of Lebanese, 38.7% of Bahraini are obese. Whereas, the proportion of overweight is 46.5% among the Lebanese (23) and 39.7% among the Bahraini (24). This trend of increased obesity and overweight in our study is mostly attributable to modern lifestyle changes, such as easy transportation, long office hours, and hot weather conditions, in addition to many other social factors that together inhibit healthy physical activities of walking, jogging, or outdoor playing among the Saudi population.

The prevalence of obesity as reported in the two studies in systematic Review among women in Saudi Arabia was 57.1 and 46.7%, respectively (25). Based on the data from the Framingham Offspring project, obesity, as calculated by BMI markedly and independently, influences the incidence of CHD and CVA after adjusting for the traditional risk factors (26). The higher WC of more than 88 cm among 78% of them may be due to the unhealthy diet, lack of exercise, and maintenance of gestational weight gain. This number was more than the reported percentage among the female employees in King Faisal University in Al Hassa, Saudi Arabia, where the mean WC was 91.7 ± 16.5 cm (*CI* = 89.4–101.8), 46.5% had a WC >88 cm, and 38.4% were overweight (27).

In this study, the most reported chronic disease was HTN, i.e., 53%, and that is higher than that of Lebanese 29.8% (23), Bahrainis 36.9% (24), Kuwaitis 8.9%(28), and out of a systematic review among the Saudi was 21.8% (25). This higher prevalence of HTN may be due to the inclusion of older participants in our study.

Unfortunately, HTN is a well-established risk factor for adverse cardiovascular outcomes, such as deaths from CHD and stroke (29). In a cohort of over 1.25 million patients aged 30 years and above without baseline CVD, including 20% with baseline treated HTN, the hypertensive patients had a 63.3% lifetime risk of developing CVD compared with a 46.1% risk for those with normal baseline BP (1). Regarding diabetes among our sample, the percentage was 7.2% compared with 6.6% of Kuwaitis (28), 22.8% in a study of Lebanese (23), and a range between 4 and 5.2% among the Saudi women, according to a systematic review (25). Patients with T2DM have 2–4 times increase in the risk of incident CHD and ischemic stroke and 1.5 to 3.6-fold increase in mortality (30). Regardless of the estimated 10-year ASCVD risk, it is recommended to treat T2DM patients who are between 40 and 75 years of age with a moderate-intensity statin drug. For patients with various ASCVD risk factors, the high-intensity statin drugs are favorable for decreasing the LDL-C levels by 50% or more.

As we expected based on the data regarding BMIs and abdominal obesity of our participants, 85% of them regularly consumed an unhealthy diet. Overall knowledge of study participants was found to be 8.6, that is considered as low knowledge. Subsequently, that would lead to delay in seeking medical care, impacting negatively to the patients' outcomes. This is in line with the problem of other studies conducting in different populations worldwide, the majority of Jordanian participants were found to have poor to moderate knowledge of CVDs (31), and Kuwaiti respondents had poor knowledge of CVD types, i.e., both stroke and heart attack symptoms, but moderate knowledge of CVD risk factors (28). Muhamad et al. found limited knowledge and practice among the female patients in primary care facilities in Malaysia (32), while Fahs et al. found good knowledge of CVD risk factors among the Lebanese population (23). Concerning stroke risk factors and warning symptoms, a study of Saudis by Alreshidi et al. found that 63.8% of their participants had insufficient knowledge, attitudes, and practice (33). A second study by Alhazzani also showed a deficit in knowledge about strokes (34). The perception of individual oneself at higher risk increases when the existence of a risk factor is already known. The implementation of awareness programs targeting the population in attractive and convincing ways is a must to increase knowledge and awareness. Furthermore, the most reported stroke symptoms in this study were sudden dizziness and difficulty walking/loss of balance, reported by 63%, whereas in Awad's study (28), sudden confusion and trouble speaking or understanding others was most prevalent. In our study, like the Jordanian study (30), chest pain or discomfort was the most reported heart attack symptom 78%, and 82% reported that higher than the percentage in the Kuwaiti study 50% (28).

The results showed a higher awareness of unhealthy diet [85%], HTN [80%], obesity [79%], smoking [77%], dyslipidemia [75%], and lack of exercise [71%] as CVD risk factors. This is mainly because of the high prevalence of obesity and HTN among Saudi population who receive frequent non-pharmacological advice by healthcare providers to link these factors as contributors to CVD morbidity and mortality in their daily clinic.

Among the Kuwaiti cohort (28), tobacco, obesity, unhealthy food, and physical inactivity were commonly reported, while hypercholesterolemia, HTN, diabetes mellitus, stress, and a family history of CVD were less frequently identified as the CHD risk factors. Smoking was the most commonly reported risk factor [75.7%], followed by obesity [71.2%] and a high-fat diet [62.0%], among the Jordanian population (31). The study of the Lebanese population showed that they were most aware of smoking as a CVD risk factor and least aware of diabetes (23). This may be due to countries' variations in the prevalence of public health problems and information provided by the mass media.

In addition, in this study, the elements independently associated with CVD knowledge were age, education, smoking, consuming healthy food, and family history of CVD (*p* < 0.05).

Knowledge of CVD was considerably greater among those aged 40–51 years compared with other age groups. The study participants were observed to be more knowledgeable of CVD if they reported high education levels, healthy daily diets, and a family history of CVD. So, education is considered as an important element to increase the public health and improving the quality of life.

In the Kuwaiti study, the independent factors related to a good level of CVD knowledge were, among women aged 50–59 years, a high degree of learning, consumption of healthy food, and a family history of CVD (28). The evidence from the studies showed that the degree of education is a potent predictor of CVD knowledge as those who had obtained higher education had better CVD knowledge scores (31). Good attitude and practice were significantly associated with younger age groups and highly educated participants (33). Our study found that the women who reported having a family history of CVD had a good knowledge percentage compared with the participants without such a history, which is similar to the findings of Mukattash et al. (31) and Al Hamarneh et al. (35). This is because their awareness increases through sharing of the experiences by their family members.

The results of our research have revealed that in 13.5 and 3.2% of women with intermediate and high risks of CVD, there is a strong association between the increase in the risk of CVD and the increase in age, BMI, blood pressure, and cholesterol levels. One-quarter of the 4,500 participants in Alzeidan's study were found to have a >10% risk of suffering CVD within the next 10 years and so not only increasing morbidity and mortality but also increasing demands of tertiary healthcare (36). Another figure from Saudi research shows that among 4,932 individuals, 55% of whom were female, the risk of coronary disease within the next 10 years was low in 92.6%, intermediate in 3.2%, and high in 4.1% of the subjects (37). In a study by Saeedi et al., in which 65% of study participants were female, 69% of the participants were in a low- risk group, while 31% were in the moderate and high-risk groups^1^. Additionally, among 2,029 women in the AlQuaiz study, 83% were low risk (Score <10), and 17% had intermediate-to-high Framingham risk scores (38). Lastly, the CVD risk factors must be well-known at national level for entire population to decrease curve of death rate related to CVD.

### Limitations and Strengths

A smaller sample size and inclusion of only female participants limit the generalizability of results. The precision of the study might be low because of the relatively small sample size, thus generalizability of the findings to the larger Saudi population was difficult. In addition, limited comparison with data from studies conducted in the Arab population with specific dietary habits and lifestyles that may consider expansion and preparing a separate paper in our future study on the CVD risk and lifestyles habit. Furthermore, the awareness and knowledge of risk factors are investigated, but not perception. On the other hand, this study addresses a pertinent issue of CVDs and their risk factors in Madinah city will add to the current knowledge of CVD in KSA and the Gulf countries, carried among an educated sample that will help later to raise the awareness of CVD risk factors in our community. The epidemiological investigation tools used are valid and reliable. The relation between dietary habits and CVD risk factors will be studied in a separate paper.

## Conclusions

Low levels of knowledge regarding CVD risk factors were reported among the participants. There was a high percentage of obesity and increased abdominal circumference among them. HTN was a significant chronic disease as more than half of the sample reported it. Frequently recognized risk factors were unhealthy diet, HTN, obesity, smoking, dyslipidemia, and lack of exercise. In this study, the factors independently associated with CVD knowledge were age, education, smoking, eating a healthy diet, and family history of CVD.

Health stakeholders may use these results for future planning of educational programs to promote the awareness of risk factors and prevention of CVD in Madinah. Primary prevention strategies can positively reduce the ASCVD risk if certain healthy behaviors are adopted early and continued throughout life, and these strategies will help people avoid developing established ASCVD risk factors, such as high BP, obesity, and hyperlipidemia. However, more studies that include larger samples of both gender from different region in Saudi kingdom are needed to validate the findings of this study.

## Data Availability Statement

The original contributions presented in the study are included in the article/supplementary material, further inquiries can be directed to the corresponding author/s. The datasets generated for this study are available on request to the corresponding author.

## Author Contributions

AQ conceptualized the idea of research based on the importance and relevance of the topic and contributed to literature search, provided research materials, collected and organized data and references, and provided logistic support. WM identified the appropriate methods of analysis, interpreted the results of the study, and wrote the final draft of the article. AS helped in enriching references and writing discussion of the study. All authors have contributed to writing, designing, critically review and so approved the final draft, and are responsible for the content included here.

## Conflict of Interest

The authors declare that the research was conducted in the absence of any commercial or financial relationships that could be construed as a potential conflict of interest.

## Publisher's Note

All claims expressed in this article are solely those of the authors and do not necessarily represent those of their affiliated organizations, or those of the publisher, the editors and the reviewers. Any product that may be evaluated in this article, or claim that may be made by its manufacturer, is not guaranteed or endorsed by the publisher.

## Footnotes

^1^ https://journalissues.org/wp-content/uploads/2018/03/Saeedi-et-al.pdf

## Abbreviations

FBG: fasting blood glucose
TLCs: therapeutic lifestyle changes
CVD: cardiovascular disease
CAD: coronary artery disease
ASCVD: atherosclerotic cardiovascular disease
LDL: low density lipoprotein
TC: total cholesterol
BMI: body mass index
NCD: non-communicable disease
BP: blood pressure
CHD: coronary heart disease
IFG: impaired fasting glucose
CVA: cerebrovascular accident
IGT: impaired glucose tolerance
T2DM: type 2 diabetes mellitus
CVA: cerebrovascular accident.
